# Crystallographic and spectroscopic characterization of 3-chloro-5-fluoro­salicyl­aldehyde

**DOI:** 10.1107/S2056989020014425

**Published:** 2020-11-03

**Authors:** Christopher T. Triggs, Joseph M. Tanski

**Affiliations:** a Department of Chemistry, Vassar College, Poughkeepsie, NY 12604, USA

**Keywords:** crystal structure, hydrogen bonding, halogenated salicyl­aldehyde derivative

## Abstract

The title compound (systematic name: 3-chloro-5-fluoro-2-hy­droxy­benzaldehyde), C_7_H_4_ClFO_2_, is a dihalogenated salicyl­aldehyde derivative that has been studied for its anti­bacterial characteristics. The salicyl­aldehyde engages in intra­molecular hydrogen bonding with an O—H⋯O distance of 2.6231 (19) Å while the mol­ecules pack together *via* weak inter­molecular C—H⋯O, C—H⋯F and F⋯O inter­actions and offset face-to-face π-stacking.

## Abstract 2

The title dihalogenated salicyl­aldehyde derivative has been studied for its anti­bacterial characteristics. The salicyl­aldehyde engages in intra­molecular hydrogen bonding at a distance of 2.6231 (19) Å while the mol­ecules pack together *via* weak inter­molecular C—H⋯O, C—H⋯F and F⋯O inter­actions and offset face-to-face π-stacking.

## Chemical context   

Salicyl­aldehyde and its derivatives, including the title compound 3-chloro-5-fluoro-2-hy­droxy­benzaldehyde, (I), play an important role in the synthesis of novel anti­microbial complexes (Bozkır *et al.*, 2012 ▸; Dahlgren *et al.*, 2010 ▸; Sarı *et al.*, 2013 ▸). The title compound, commonly known as 3-chloro-5-fluoro­salicyl­aldehyde, may be synthesized by the formyl­ation of 2-chloro-4-fluoro­phenol with chloro­form through reflux with concentrated NaOH(aq) (Balko *et al.*, 2007 ▸).
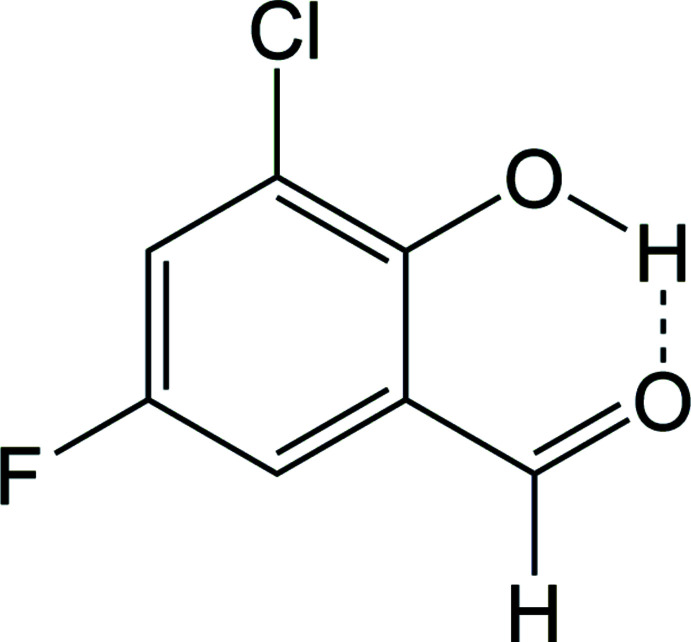


## Structural commentary   

**Figure 1:**
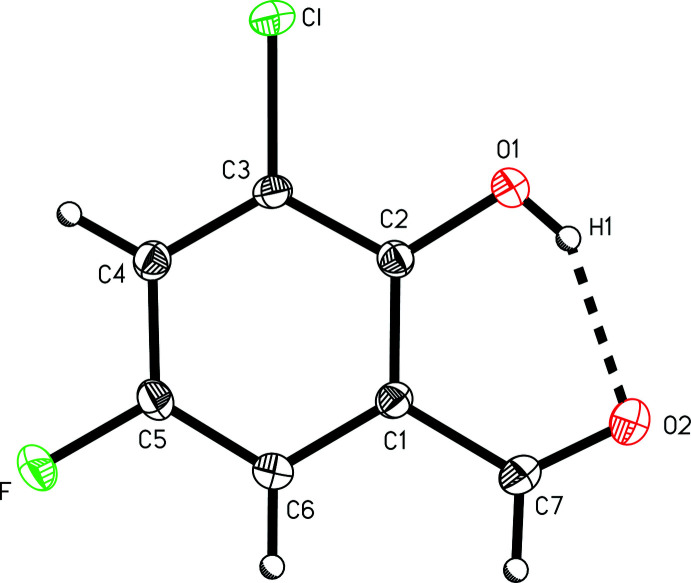
A view of 3-chloro-5-fluoro­salicyl­aldehyde, (I), depicting the intra­molecular hydrogen bonding with the atom-numbering scheme. Dis­place­ment ellipsoids are shown at the 50% probability level.

The mol­ecular structure of the title compound (Fig. 1 ▸) is planar, with an r.m.s. deviation from the plane of all non-hydrogen atoms of 0.0135 Å. The mol­ecule engages in intra­molecular hydrogen bonding between the phenol hydrogen atom and formyl functional group oxygen with an O1⋯O2 distance of 2.6231 (19) Å characterizing the O1—H1⋯O2 inter­action. The C3—Cl and C5—F bond lengths were found to be 1.7334 (16) and 1.3529 (19) Å, respectively.

## Supra­molecular features   

**Figure 2:**
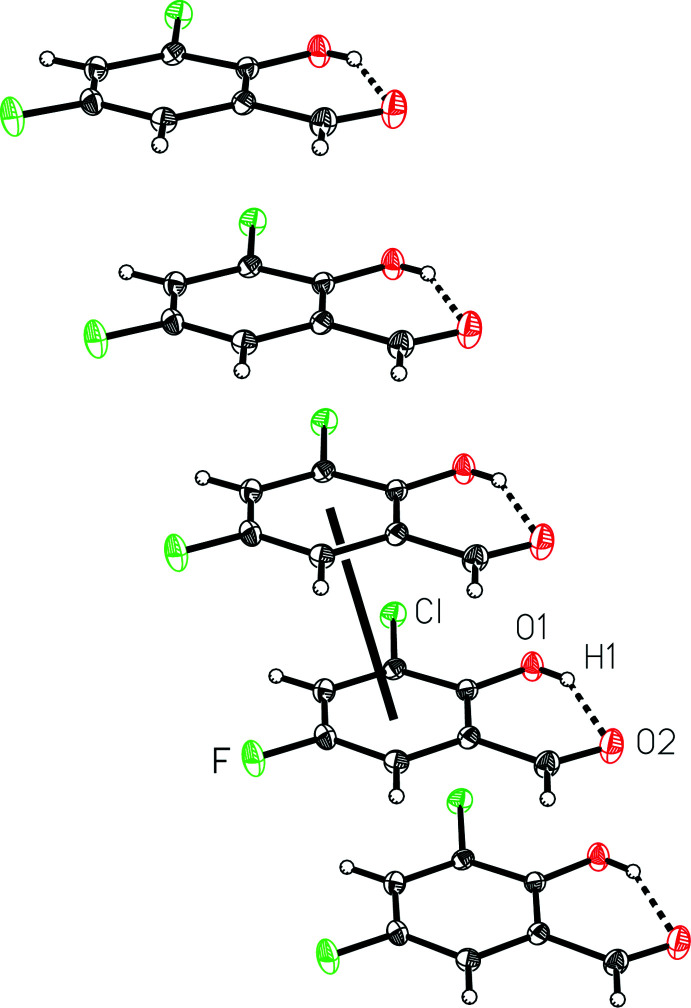
A view of the inter­molecular π-stacking in 3-chloro-5-fluoro­salicyl­aldehyde, (I), with the thick line indicating a centroid-to-centroid relationship.

**Figure 3:**
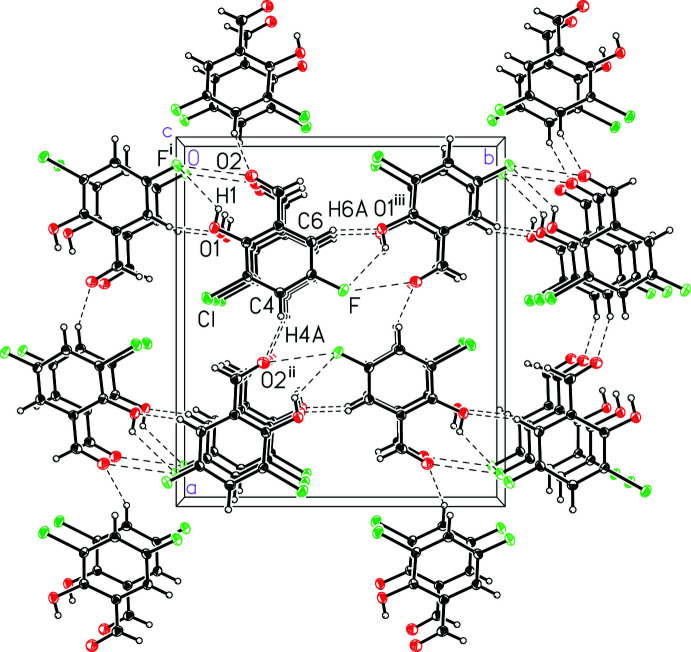
A view of the packing and weak inter­molecular C—H⋯O, C—H⋯F and F⋯O inter­actions in 3-chloro-5-fluoro­salicyl­aldehyde, (I). Symmetry codes are defined in Table 1 ▸.

The mol­ecules pack together in the solid state with weak inter­molecular C—H⋯O, C—H⋯F and F⋯O inter­actions and an offset face-to-face π-stacking geometrical relationship. Mol­ecules of 3-chloro-5-fluoro­salicyl­aldehyde form one-dimensional π-stacking chains (Fig. 2 ▸), which are characterized by a ring centroid-to-centroid distance of 3.7154 (3) Å, a centroid-to-plane distance of 3.3989 (8) Å, and a ring-offset slippage of 1.501 (2) Å. These π-stacking chains are linked together to form the three-dimensional structure through weak inter­molecular C—H⋯O and C—H⋯F contacts (Table 1 ▸ and Fig. 3 ▸). Specifically, O1—H1⋯F^i^, C4—H4*A*⋯O2^ii^, and C6—H6*A*⋯O1^iii^ inter­molecular inter­actions (symmetry codes as defined in Table 1 ▸), with donor–acceptor distances of 3.0101 (18), 3.254 (2) and 3.377 (2) Å, respectively. In addition, an O2⋯F^i^ contact with a distance of 2.880 (2) Å is observed. Notably, there are no close halogen–halogen inter­actions.

## Database survey   

The Cambridge Structural Database (Version 5.40, update of March 2020; Groom *et al.*, 2016 ▸) contains many halogenated benzene structures and relatively few halogenated salicyl­aldehyde structures. Literature aryl C—Cl and C—F bond lengths, as seen in halogenated benzene crystal structures, are similar to those seen in the title compound. For example, the C—Cl distances in 1,2- and 1,3-di­chloro­benzene range between 1.731 (3) and 1.756 (3) Å (ABUMIT and ABUMOZ; Boese *et al.*, 2001 ▸), while the C—F distances in 1,3-di­fluoro­benzene are found to be between 1.3486 (14) and 1.3553 (13) Å (PUGDAX; Kirchner *et al.*, 2009 ▸). Related salicyl­aldehyde structures that differ in the number of halogen atoms include unsubstituted salicyl­aldehyde itself (YADJOE; Kirchner *et al.*, 2011 ▸), 5-chloro­salicyl­aldehyde (RAJGOA01; Aitken *et al.*, 2013 ▸; RAJGOA, Jin *et al.*, 2011 ▸), and 3,5-di­chloro­salicyl­aldehyde (MIXYEY; Azizul & Ng, 2008 ▸). As with the title compound, each of these structures exhibits strong intra­molecular hydrogen bonding between the phenol hydrogen atom and formyl oxygen atom.

## Synthesis and crystallization   

3-Chloro-5-fluoro­salicyl­aldehyde (I, 97%) was purchased from Aldrich Chemical Company, USA, and was used as received.

## Refinement   

Crystal data, data collection and structure refinement details are summarized in Table 2 ▸. All non-hydrogen atoms were refined anisotropically. Hydrogen atoms bonded to carbon were included in calculated positions and refined using a riding model with C—H = 0.95 and *U*_iso_(H) = 1.2*U*_eq_(C) for the aryl H atoms. The position of the phenolic hydrogen atom was found in the difference map and refined freely.

## Analytical Data   

^1^H NMR (Bruker Avance III 400 MHz, CDCl_3_): δ 7.23 (*dd*, 1H, C_ar­yl_ *H, J_meta_* = 3.0 Hz, *J*_H–F_ = 7.2 Hz), 7.42 (*dd*, 1H, C_ar­yl_ *H, J_meta_* = 3.0 Hz, *J*_H–F_ = 7.8 Hz), 9.86 (*s*, 1H, O*H*), 11.21 (*s*, 1H, C(=O)*H*). ^13^C NMR (^13^C{^1^H}, 100.6 MHz, CDCl_3_): δ 116.90 (*d*, *C*_ar­yl_H, *J*_C–F_ = 22.6 Hz), 120.24 (*d*, *C*_ar­yl_, *J*_C–F_ = 6.6 Hz), 123.15 (*d*, *C*_ar­yl_, *J*_C–F_ = 9.1 Hz), 124.72 (*d*, *C*_ar­yl_H, *J*_C–F_ = 26.4 Hz), 153.85 (*d*, *C*_ar­yl_, *J*_C–F_ = 2.2 Hz), 154.85 (*d*, *C*_ar­yl_F, *J*_C–F_ = 244.0 Hz), 194.97 (*C*(=O)H). ^19^F NMR (^19^F{^1^H}, 376.5 MHz, CDCl_3_): δ −121.50. IR (Thermo Nicolet iS50, ATR, cm^−1^) : 3081 (*m br*, O—H & C_ar­yl_—H *str*), 2859 [*w*, C(=O)—H *fermi doublet str*], 2733 [*w*, C(=O)—H *fermi doublet str*], 1803 (*w*), 1754 (*w*), 1664 (*s*, C=O *str*), 1623 (*m*), 1583 (*w*), 1524 (*w*), 1462 (*m*), 1436 (*s*), 1373 (*m*), 1351 (*w*), 1294 (*s*), 1238 (*s*), 1183 (*s*), 1119 (*s*), 982 (*s*), 902 (*m*), 894 (*m*), 875 (*s*), 802 (*s*), 727 (*s*), 708 (*s*), 578 (*m*), 530 (*s*), 493 (*s*), 458 (*m*). GC/MS (Agilent MS 5975/GC 7890): *M* ^+^ = 174 (calc. exact mass 173.99).

## Supplementary Material

Crystal structure: contains datablock(s) global, I. DOI: 10.1107/S2056989020014425/pk2651sup1.cif

Structure factors: contains datablock(s) I. DOI: 10.1107/S2056989020014425/pk2651Isup2.hkl

Supporting information file. DOI: 10.1107/S2056989020014425/pk2651Isup3.cml

CCDC reference: 2041401

Additional supporting information:  crystallographic information; 3D view; checkCIF report

## Figures and Tables

**Table 1:** Hydrogen-bond geometry (Å, °)

*D*—H⋯*A*	*D*—H	H⋯*A*	*D*⋯*A*	*D*—H⋯*A*
O1—H1⋯F^i^	0.77 (3)	2.47 (3)	3.0101 (18)	128 (2)
O1—H1⋯O2	0.77 (3)	1.93 (3)	2.6231 (19)	150 (3)
C4—H4*A*⋯O2^ii^	0.95	2.37	3.254 (2)	155
C6—H6*A*⋯O1^iii^	0.95	2.55	3.377 (2)	145

Symmetry codes: (i) 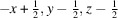; (ii) 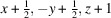; (iii) 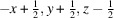.

**Table 2:** Experimental details

Crystal data	
Chemical formula	C_7_H_4_ClFO_2_
*M*_r_	174.55
Crystal system, space group	Orthorhombic, *P* *n* *a*2_1_
Temperature (K)	125
*a*, *b*, *c* (Å)	14.2730 (13), 12.7102 (12), 3.7154 (3)
*V* (Å^3^)	674.02 (10)
*Z*	4
Radiation type	Mo *K*α
μ (mm^−1^)	0.52
Crystal size (mm)	0.45 × 0.10 × 0.02

Data collection	
Diffractometer	Bruker APEXII CCD
Absorption correction	Multi-scan (*SADABS*; Bruker, 2017 ▸)
*T*_min_, *T*_max_	0.87, 0.99
No. of measured, independent and observed [*I* > 2σ(*I*)] reflections	16131, 2052, 1985
*R*_int_	0.030
(sin θ/λ)_max_ (Å^−1^)	0.715

Refinement	
*R*[*F* ^2^ > 2σ(*F* ^2^)], *wR*(*F* ^2^), *S*	0.023, 0.066, 1.07
No. of reflections	2052
No. of parameters	104
No. of restraints	1
H-atom treatment	H atoms treated by a mixture of independent and constrained refinement
Δρ_max_, Δρ_min_ (e Å^−3^)	0.34, −0.19
Absolute structure	Flack *x* determined using 837 quotients [(*I* ^+^)−(*I* ^−^)]/[(*I* ^+^)+(*I* ^−^)] (Parsons *et al.*, 2013 ▸)
Absolute structure parameter	0.07 (2)

Computer programs: *APEX2* and *SAINT* (Bruker, 2017 ▸), *SHELXT2014/5* (Sheldrick, 2015*a* ▸), *SHELXL2017/1* (Sheldrick, 2015*b* ▸), *SHELXTL* (Sheldrick, 2008 ▸), *OLEX2* (Dolomanov *et al.*, 2009 ▸), and *Mercury* (Macrae *et al.*, 2020 ▸).

## supplementary crystallographic information

### Crystal data

**Table:**

C_7_H_4_ClFO_2_	*D*_x_ = 1.720 Mg m^−^^3^
*M**_r_* = 174.55	Mo *K*α radiation, λ = 0.71073 Å
Orthorhombic, *P**n**a*2_1_	Cell parameters from 9931 reflections
*a* = 14.2730 (13) Å	θ = 2.9–30.5°
*b* = 12.7102 (12) Å	µ = 0.52 mm^−^^1^
*c* = 3.7154 (3) Å	*T* = 125 K
*V* = 674.02 (10) Å^3^	Needle, colourless
*Z* = 4	0.45 × 0.10 × 0.02 mm
*F*(000) = 352	

### Data collection

**Table:**

Bruker APEXII CCD diffractometer	2052 independent reflections
Radiation source: fine-focus sealed tube	1985 reflections with *I* > 2σ(*I*)
Graphite monochromator	*R*_int_ = 0.030
Detector resolution: 8.3333 pixels mm^-1^	θ_max_ = 30.5°, θ_min_ = 2.2°
φ and ω scans	*h* = −20→20
Absorption correction: multi-scan (SADABS; Bruker, 2017)	*k* = −18→17
*T*_min_ = 0.87, *T*_max_ = 0.99	*l* = −5→5
16131 measured reflections	

### Refinement

**Table:**

Refinement on *F*^2^	Secondary atom site location: difference Fourier map
Least-squares matrix: full	Hydrogen site location: mixed
*R*[*F*^2^ > 2σ(*F*^2^)] = 0.023	H atoms treated by a mixture of independent and constrained refinement
*wR*(*F*^2^) = 0.066	*w* = 1/[σ^2^(*F*_o_^2^) + (0.0394*P*)^2^ + 0.1224*P*] where *P* = (*F*_o_^2^ + 2*F*_c_^2^)/3
*S* = 1.07	(Δ/σ)_max_ < 0.001
2052 reflections	Δρ_max_ = 0.34 e Å^−^^3^
104 parameters	Δρ_min_ = −0.19 e Å^−^^3^
1 restraint	Absolute structure: Flack *x* determined using 837 quotients [(*I*^+^)-(*I*^-^)]/[(*I*^+^)+(*I*^-^)] (Parsons *et al.*, 2013)
Primary atom site location: dual	Absolute structure parameter: 0.07 (2)

### Special details

**Table:**

Geometry. All esds (except the esd in the dihedral angle between two l.s. planes) are estimated using the full covariance matrix. The cell esds are taken into account individually in the estimation of esds in distances, angles and torsion angles; correlations between esds in cell parameters are only used when they are defined by crystal symmetry. An approximate (isotropic) treatment of cell esds is used for estimating esds involving l.s. planes.

### Fractional atomic coordinates and isotropic or equivalent isotropic displacement parameters (Å^2^)

**Table:**

	*x*	*y*	*z*	*U*_iso_*/*U*_eq_	
Cl	0.43892 (3)	0.11149 (3)	0.78847 (17)	0.02049 (11)	
F	0.41791 (7)	0.50784 (8)	0.7158 (3)	0.0246 (3)	
O1	0.25167 (10)	0.12611 (10)	0.4770 (4)	0.0204 (3)	
H1	0.202 (2)	0.137 (2)	0.412 (8)	0.036 (8)*	
O2	0.11001 (9)	0.23221 (11)	0.2114 (4)	0.0256 (3)	
C1	0.24600 (12)	0.31544 (12)	0.4365 (4)	0.0143 (3)	
C2	0.29149 (11)	0.22104 (12)	0.5277 (4)	0.0142 (3)	
C3	0.38146 (11)	0.22705 (12)	0.6760 (4)	0.0141 (3)	
C4	0.42463 (11)	0.32301 (13)	0.7379 (4)	0.0157 (3)	
H4A	0.485639	0.326265	0.8395	0.019*	
C5	0.37672 (11)	0.41404 (13)	0.6482 (5)	0.0160 (3)	
C6	0.28892 (11)	0.41307 (13)	0.4973 (4)	0.0157 (3)	
H6A	0.258084	0.476816	0.435752	0.019*	
C7	0.15263 (10)	0.31310 (13)	0.2738 (6)	0.0186 (3)	
H7A	0.123791	0.378134	0.213767	0.022*	

### Atomic displacement parameters (Å^2^)

**Table:**

	*U*^11^	*U*^22^	*U*^33^	*U*^12^	*U*^13^	*U*^23^
Cl	0.02085 (18)	0.01654 (18)	0.02407 (19)	0.00416 (13)	−0.00540 (17)	0.00218 (18)
F	0.0206 (5)	0.0149 (5)	0.0384 (7)	−0.0046 (4)	−0.0032 (5)	−0.0033 (5)
O1	0.0189 (6)	0.0142 (6)	0.0281 (7)	−0.0017 (5)	−0.0059 (5)	0.0005 (5)
O2	0.0192 (5)	0.0225 (6)	0.0350 (9)	−0.0014 (5)	−0.0094 (6)	−0.0006 (5)
C1	0.0138 (7)	0.0147 (7)	0.0145 (6)	0.0007 (5)	−0.0012 (5)	0.0000 (6)
C2	0.0156 (7)	0.0133 (7)	0.0136 (6)	−0.0007 (5)	−0.0007 (6)	0.0000 (6)
C3	0.0153 (7)	0.0133 (7)	0.0139 (6)	0.0024 (5)	−0.0015 (5)	0.0006 (5)
C4	0.0141 (6)	0.0165 (7)	0.0166 (8)	0.0001 (5)	−0.0005 (6)	−0.0012 (6)
C5	0.0160 (7)	0.0135 (7)	0.0185 (7)	−0.0025 (6)	0.0011 (6)	−0.0017 (6)
C6	0.0167 (7)	0.0136 (7)	0.0167 (7)	0.0011 (6)	0.0001 (6)	0.0005 (6)
C7	0.0162 (7)	0.0192 (7)	0.0205 (7)	0.0022 (5)	−0.0031 (7)	0.0008 (8)

### Geometric parameters (Å, º)

**Table:**

Cl—C3	1.7334 (16)	C2—C3	1.399 (2)
F—C5	1.3529 (19)	C3—C4	1.386 (2)
O1—C2	1.3470 (19)	C4—C5	1.385 (2)
O1—H1	0.77 (3)	C4—H4A	0.95
O2—C7	1.217 (2)	C5—C6	1.373 (2)
C1—C6	1.402 (2)	C6—H6A	0.95
C1—C2	1.406 (2)	C7—H7A	0.95
C1—C7	1.464 (2)		
			
C2—O1—H1	106 (2)	C5—C4—H4A	120.8
C6—C1—C2	120.99 (14)	C3—C4—H4A	120.8
C6—C1—C7	118.83 (14)	F—C5—C6	118.71 (15)
C2—C1—C7	120.18 (14)	F—C5—C4	118.49 (14)
O1—C2—C3	119.41 (14)	C6—C5—C4	122.79 (15)
O1—C2—C1	122.42 (14)	C5—C6—C1	118.19 (15)
C3—C2—C1	118.17 (14)	C5—C6—H6A	120.9
C4—C3—C2	121.43 (14)	C1—C6—H6A	120.9
C4—C3—Cl	119.70 (12)	O2—C7—C1	123.44 (15)
C2—C3—Cl	118.87 (12)	O2—C7—H7A	118.3
C5—C4—C3	118.42 (15)	C1—C7—H7A	118.3

### Hydrogen-bond geometry (Å, º)

**Table:**

*D*—H···*A*	*D*—H	H···*A*	*D*···*A*	*D*—H···*A*
O1—H1···F^i^	0.77 (3)	2.47 (3)	3.0101 (18)	128 (2)
O1—H1···O2	0.77 (3)	1.93 (3)	2.6231 (19)	150 (3)
C4—H4*A*···O2^ii^	0.95	2.37	3.254 (2)	155
C6—H6*A*···O1^iii^	0.95	2.55	3.377 (2)	145

Symmetry codes: (i) −*x*+1/2, *y*−1/2, *z*−1/2; (ii) *x*+1/2, −*y*+1/2, *z*+1; (iii) −*x*+1/2, *y*+1/2, *z*−1/2.
